# Creating a Simulated On-Call Scenario to Measure Stress and Improve Confidence in Medical Students

**DOI:** 10.1192/bjo.2024.427

**Published:** 2024-08-01

**Authors:** Abigail Runicles, Tawfiq Alqeisi, Megan Bradley, Sharukh Zuberi, Piers Gatenby

**Affiliations:** 1 South London and Maudsley, London, United Kingdom; 2 Royal Surrey County Hospital, Surrey, United Kingdom

## Abstract

**Aims:**

Previous literature has reported that medical students are objectively and subjectively more stressed than the general population. The transition between medical school and commencing a career as a foundation doctor can cause a significant amount of stress. The first aim was to investigate stress and anxiety and how this may impact performance, with the aim being to better understand stress in medical students about to embark on a career as a doctor. The second aim was to create a simulated 1-1 on-call shift scenario to allow final year medical students to practice the skills and improve confidence.

**Methods:**

16 final year medical students from two UK medical universities took part in a simulated on-call scenario acting as the foundation year 1 doctor. During the scenario, participants were scored on their performance. Fitbits measured heart rate data as an objective measure of stress. Subjective data was collected using the State-Trait Anxiety Inventory (STAI). They were asked a series of questions regarding their confidence before and after the scenario.

**Results:**

Participants reported higher states of anxiety after the on-call simulation compared with a regular day on placement (t=-6.93, p <0.001). There was a trend between reported higher levels of state anxiety and lower performance scores (r=-0.475, p=0.063.) There was no correlation between average heart rate and reported levels of state anxiety (r=0.452, p=0.105). Prior to the on-call scenario participants reported their confidence as follows; 26.09% no confidence, 65.22% slightly confident, 8.7% somewhat confident, 0% confident/very confident. After the scenario participants reported their confidence as follows; 4.35% no confidence, 34.78% slightly confident, 52.17% somewhat confident, confident 8.7% and 0% very confident. 100% of participants reported that they would recommend the session to colleagues.

**Conclusion:**

The results highlight that an on-call scenario has a significant impact on the feelings of stress in medical students. It also shows that stress can have a negative impact on performance. However, experience completing a simulated on-call scenario helped to improve confidence and was recommended to colleagues. Future research should aim to further investigate acute stress in a real-life setting and use objective measures of stress. Over time researchers should aim to create a targeted intervention aimed at supporting medical students and junior doctors during their on-call and provide opportunities to improve confidence.

